# Social Networks of Educated Nematodes

**DOI:** 10.1038/srep14388

**Published:** 2015-09-25

**Authors:** Denis S. Willett, Hans T. Alborn, Larry W. Duncan, Lukasz L. Stelinski

**Affiliations:** 1 University of Florida, Entomology and Nematology Department, Citrus Research and Education Center, Lake Alfred, FL, 33850, USA; 2 Agricultural Research Service, United States Department of Agriculture, Center for Medical, Agricultural and Veterinary Entomology, Gainesville, FL, 32608, USA

## Abstract

Entomopathogenic nematodes are obligate lethal parasitoids of insect larvae that navigate a chemically complex belowground environment while interacting with their insect hosts, plants, and each other. In this environment, prior exposure to volatile compounds appears to prime nematodes in a compound specific manner, increasing preference for volatiles they previously were exposed to and decreasing attraction to other volatiles. In addition, persistence of volatile exposure influences this response. Longer exposure not only increases preference, but also results in longer retention of that preference. These entomopathogenic nematodes display interspecific social behavioral plasticity; experienced nematodes influence the behavior of different species. This interspecific social behavioral plasticity suggests a mechanism for rapid adaptation of belowground communities to dynamic environments.

Social behavioral plasticity, modification and transference of behavior based on past experience, is an important adaptive response to dynamic environments that can be observed across many species with various levels of complexity. While complex adaptive behaviors have been recorded in animals with advanced cognitive circuitry (e.g. social learning in primates and New Caledonian crows^1^,^2^) the basis for these behaviors can be found in simpler organisms^3^. For example, as a model organism, the free living bacterivorous nematode *Caenorhabditis elegans*, has been used extensively to investigate both the extent and genetic basis of learning in soil microorganisms^4^. Despite only 302 neurons, *C. elegans* demonstrates behavioral plasticity by modifying behavior in response to exposure to thermal, tactile, and chemical cues^3^,^4^. In addition, under certain starvation-induced conditions, *C. elegans* demonstrates collective behavior; they aggregate^5^.

While the capacity for behavioral plasticity in nematodes has been demonstrated in *C. elegans*, the implications of such plasticity and ramifications for aggregation in an environmental and social context remain to be explored. Entomopathogenic nematodes (EPNs) are ideally suited for exploring such implications in an ecologically and agriculturally relevant way. Entomopathogenic nematodes inhabit dynamic belowground environments, are obligate parasitoids of insect larvae, and rely upon endosymbiotic bacteria to kill their host^6^. As the bacteria multiply in the insect cadaver, the EPNs feed on the bacteria, mate, and, when the host is consumed, produce an infective juvenile life stage that leaves the host in search of more insects^6^. Finding additional insect hosts can be a challenge; insect distributions are often highly clumped^7^. After exploiting resources in one location, nematodes may have to travel large distances and rely upon a variety of chemically mediated behaviors to do so. Dispersal from the host larvae is mediated by specific blends of ascarosides, dideoxysugar derivatives produced by nematodes in the insect host cadaver^8^. During dispersal, EPNs respond to herbivore induced plant volatiles released by plant roots^9^. For example, in maize, feeding by the beetle *Diabrotica virgifera virgifera* results in release of volatile compounds, among them, E-*β* caryophyllene which recruits the EPN *Heterorhabditis megidis*^10^. In certain citrus cultivars, larval feeding on roots by the weevil *Diaprepes abbreviatus* results in the release of pregeijerene, which not only is highly attractive to a variety of EPN species, but also recruits free living and plant parasitic nematodes^11^,^12^.

In addition to these herbivore induced plant volatiles, EPNs in citrus must navigate a myriad of additional stimuli including additional volatiles released by plants^9^,^11^ and another critical component of the belowground environment—themselves. Entomopathogenic nematodes remain in close proximity to large numbers of related individuals throughout much of their lifecycle, emerging from host cadavers in large numbers, aggregating in the environment^13^,^14^,^15^,^16^,^17^, and relying upon one another to successfully overcome the defenses of the host insect^18^. Given their dynamic belowground environment, entomopathogenic nematodes may benefit from volatile recognition (behavioral plasticity after volatile exposure), remembering persistent signals (learning and memory), and communication of past experience (interspecific social behavioral plasticity).

## Results

### Behavioral Plasticity after Volatile Exposure

**Figure 1:**
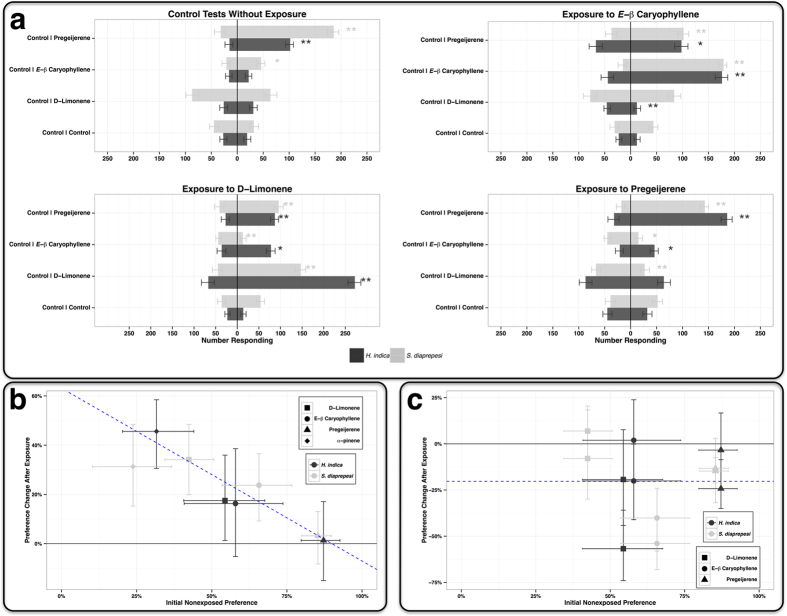
Behavioral plasticity trials using two choice olfactometer assays with exposed and nonexposed infective juveniles. (**a**) Responses of exposed and nonexposed infective juvenile entomopathogenic nematodes to belowground volatiles. Bars denote mean number of respondents; error bars denote standard error. Single asterisks denote significance (*P* < 0.05) by the exact binomial test. Double asterisks denote high significance (*P* < 0.001) by the exact binomial test. (**b**) Behavioral plasticity in trials where assayed treatment matched exposure (e.g. EPN IJs were exposed to D-limonene then assayed against D-limonene). Initial nonexposed treatment preference (horizontal axis) is the percentage of nonexposed IJs responding to a compound in two choice bioassays. Behavioral change after exposure (vertical axis) is the difference between preference of exposed IJs for the same compound and initial treatment preference of nonexposed IJs. Blue dashed line is the bootstrapped relationship across species and compounds between change after exposure and nonexposed treatment preference. Change in preference after exposure decreased 7% (95% CI: 5.7%, 8.4%) with every 10% increase in initial nonexposed preference. (**c**) Behavioral plasticity in trials where the nematodes were exposed to one of the three compounds and the assayed treatment did not match exposure (e.g. EPN IJs were exposed to D-limonene then assayed against E-*β* caryophyllene and pregeijerene). Axes are as in 1b, but note different vertical scale. Blue dashed line is the bootstrapped mean change after exposure; there was no significant (*P* = 0.89) linear relationship between change after exposure and nonexposed treatment preference. Treatment preference for a given compound declined on average by 20.3% (15%, 25.4%) after exposure to other compounds.

Using two choice, sand-filled olfactometers, we assayed preferences of the entomopathogenic nematodes *Steinernema diaprepesi* and *Heterorrhabtidis indica* to volatiles commonly present in the citrus rhizosphere both before and after exposure to those volatiles (Fig. 1). Prior to exposure, both species did not display significant (*P* > 0.05, exact binomial test) preferences for E-*β* caryophyllene or D-limonene, but did innately significantly prefer pregeijerene (*P* < 0.001, exact binomial test; Fig. 1a). Exposure to volatiles altered behavior: exposure to E-*β* caryophyllene resulted in preference (*P* < 0.001, exact binomial test) for E-*β* caryophyllene for both species and increased E-*β* caryophyllene preference by 23.7% (95% CI: 2.2%, 45.2%; *P* = 0.036, two sample t-test) in *S. diaprepesi*. Exposure to D-limonene resulted in preference for D-limonene for both species (*P* < 0.001, exact binomial test) and increased D-limonene preference by 34.6% (10.8%, 58.3%; *P* = 0.016, two sample t-test) in *S. diaprepesi*.

Indeed, change in preference for a given volatile after exposure to that volatile is linearly dependent on initial nonexposed preference for that volatile; change in preference after exposure decreased 7% (5.7%, 8.4%) with every 10% increase in initial nonexposed preference (Fig. 1b). If nematodes had a high intrinsic preference for a volatile, exposure to that volatile had little effect on behavior. Change after exposure was least (*H. indica*: 1.3% [−15%, 17.1%]; *S. diaprepesi*: 3.2% [−8.2%, 13%]) and not significantly different from zero (at *α* = 0.05) for pregeijerene which was initially highly attractive to nematodes (Fig. 1b). However, if nematodes were intrinsically repelled by a volatile (e.g. *α*-pinene), exposure to that volatile resulted in a large positive behavioral change. Change after exposure was greatest for *α*-pinene (*H. indica*: 45.7% [32.4%, 58.5%]; *S. diaprepesi*: 32.2% [15.9%, 49.4%]), which was initially repellent to nonexposed nematodes (Fig. 1b).

Interestingly, exposure altered preference for other volatiles to which the nematodes had not been exposed. Although there was no significant (*P* = 0.889) linear relationship between change after exposure and nonexposed treatment preference in assays where the volatile did not match exposure (e.g. EPN IJs were exposed to D-limonene then assayed against E-*β* caryophyllene), treatment preference for a given compound declined on average by 20.3% [15%, 25.4%] after exposure to other compounds (Fig. 1c). The behavioral plasticity observed in these EPNs suggests that they can not only recognize, adapt, and respond to specific volatile cues in their environment, but also that their learning has specificity. That specificity has consequences. It appears to increase preference for exposed volatiles while decreasing preference for volatiles to which EPNs had not been exposed.

### Learning and Memory

While the ability to demonstrate specific behavioral plasticity in response to volatile exposure is important for adapting to dynamic environments, the rapidity with which nematodes learn and the degree to which they remember can shed light on their ability to respond to persistent and ephemeral signals in their environment. To quantify the effect of the interaction between exposure time (learning time) and postexposure time (memory) on volatile preference, we used a D-optimal exposure x postexposure response surface design sufficient to detect quadratic curvature for both factors. As dictated by the experimental design (Table 1), *S. diaprepesi* infective juveniles were exposed from 1 to 48 hours (exposure time) to 1 *μg*/*ml α*-pinene, then washed and placed in water for an additional 1 to 48 hours (postexposure time) prior to use in two choice behavior assays with *α*-pinene vs. a blank control. A preference index (*I*_*p*_; the ratio between number of IJs responding to *α*-pinene and the number responding to a blank control; repellence: *I*_*p*_ < 1; preference *I*_*p*_ > 1) was constructed for each assay to facilitate comparisons between assays and to better resolve *α*-pinene preference.

**Figure 2:**
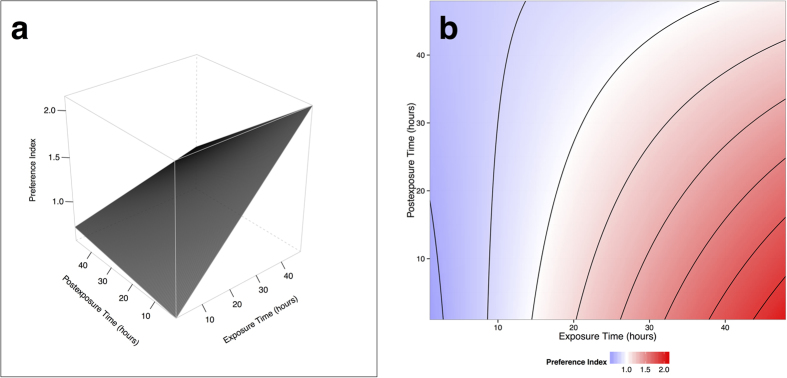
Learning and Memory Trials: the effect of exposure time, postexposure time, and their interaction on behavioral plasticity in *S. diaprepesi* (experimental design: Table 1; ANOVA and model diagnostics: Table 2). Preference index (*I*_*p*_) for *α*- pinene is constructed as the ratio of *S. diaprepesi* infective juveniles responding to *α*-pinene to those responding to blank arms in two choice olfactometers. Exposure is the amount of time in hours infective juveniles were kept in vials containing 1 *μg*/*ml α*-pinene. Postexposure is the amount of time in hours infective juveniles were kept in clean culture (i.e. plain water without additional chemical stimulus) after exposure and washing. (**a)** Preference index response surface. (**b)** Preference index contours.

Both exposure time, postexposure time, and their interaction quantifiably affected behavioral plasticity (Table 2). At low levels of exposure, *α*-pinene was repellent (*I*_*p*_ < 1) and not affected by increasing postexposure time. At high levels of exposure, *α*-pinene was highly attractive (*I*_*p*_ > 1) at low postexposure time, but preference declined with increasing postexposure time (Fig. 2). The ability to both gain and lose preference for exposed volatiles over a period of 48 hours suggests that nematodes are able to respond to relatively ephemeral cues in their environment. However, the interaction between exposure and postexposure time suggests some ability to differentiate important signals; persistent exposure results in long-term memory.

### Interspecific Social Behavioral Plasticity

**Figure 3:**
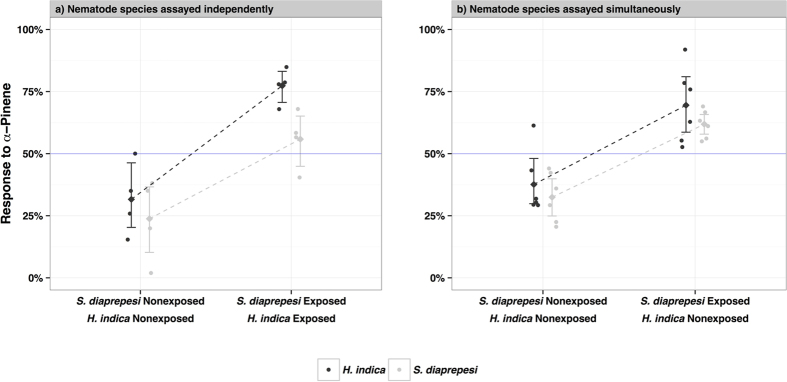
Social learning in entomopathogenic nematodes. (**a**) In independent trials, both nonexposed and exposed (to 1 *μg*/*ml α*-pinene for 48 hours) *S. diaprepesi* and *H. indica* IJs were assayed in separate two choice olfactometers against *α*-pinene. (**b**) In simultaneous trials, 2500 nonexposed *H. indica* IJs were assayed with either 100 nonexposed or 100 exposed *S. diaprepesi* IJs in the same two choice olfactometer. Points indicate the percent of recovered nematodes responding to *α*-pinene. Central diamonds and error bars denote mean percent responses and 95% confidence intervals respectively.

To determine the extent of social behavioral plasticity in entomopathogenic nematodes, we first assayed *α*-pinene preferences for both nonexposed and exposed (to 1 *μg*/*ml α*-pinene for 48 hours) *S. diaprepesi* and *H. indica* independently in two choice, sand-filled olfactometers (Fig. 3a). We then combined either 100 nonexposed or 100 exposed *S. diaprepesi* IJs simultaneously with 2500 nonexposed *H. indica* (Fig. 3b). In independent trials, *α*-pinene was initially repellent (treatment preference <0; *H. indica*: *P* = 0.08, *S. diaprepesi*: *P* = 0.05; one-sample t-test). Exposure to *α*-pinene reversed repellency and increased preference for the exposed volatile (*S. diaprepesi*: 32% [6.6%, 57.5%] increase, *P* = 0.02; *H. indica*: 45% [23.8%, 67.8%] increase, *P* = 0.003; Fig. 3a). In combined simultaneous trials, nonexposed *S. diaprepesi* and nonexposed *H. indica* were repelled by *α*-pinene (treatment preference <0; *H. indica*: *P* = 0.06, *S. diaprepesi*: *P* = 0.007; one-sample t-test). However, when exposed *S. diaprepesi* were paired with nonexposed *H. indica*, both species exhibited a preference for *α*-pinene (treatment preference >0; *H. indica*: *P* = 0.02, *S. diaprepesi*: *P* = 0.004; one-sample t-test; Fig. 3b). Nonexposed *H. indica* altered their preference for *α*-pinene when paired with exposed *S. diaprepesi*; there was social transference of behavioral plasticity from exposed to nonexposed cohorts.

## Discussion

The social transference of behavioral plasticity, not only from exposed to nonexposed cohorts but also across entomopathogenic nematode species, suggests a broad role for social behavioral plasticity in belowground communities. Individual experiences can affect future behavior on the part of an individual. As individuals learn to prefer certain signals at the expense of others while retaining and losing that preference over time, the ability to transfer that experience interspecifically suggests a mechanism for rapid adaptation of communities to dynamic belowground environments. This ability to rapidly learn based not only on past experience, but also from individuals of other species affords communities an adaptive response not available through genetic control; the existence of such rapidly adaptive behavior may suggest selection for such traits in an environment with ephemeral signals^19^.

Indeed, the adaptive ability of communities in dynamic environments may be embedded in social networks. The existence of interspecific communication between nematodes opens a realm of possibilities for using social network theory to enhance our understanding of adaptation in belowground environments. Social network structure, information flow between nematodes, and the strength of connections between individuals and species can be examined in the context of social network theory as affected by predator-prey interactions and environmental cues^20^. Integrating community adaption, nematode behavior, and social network theory may hold diverse lessons not only for belowground environments, but also for dynamic environments more generally.

Social network theory also provides a context for examining contribution of individuals to group behavior; differential contributions may be evident in the interspecific transfer of experience in entomopathogenic nematodes. This transference of learning is possibly mediated by inter-specific pheromone signals released by mobile nematodes that are used in a 'follow the leader' manner among individuals and species. Modeling suggests that follow the leader behavior could explain observed aggregative movement and arise from natural variation in nematode populations^21^. In other words, when some nematodes learn and start following a specific cue, others may follow the leaders using another cue. This may be exploited for practical management of subterranean insect pests of crops. It has been suggested that semiochemical cues that attract EPNs may be exploited for management of root-zone pests of agricultural crops with biological control^10^. The current data suggest that this tactic could be more effective than previously thought given the social transfer of learned behavior between multiple nematode species. An attractant for one EPN species may work quite broadly for general attraction of commensal species. This could possibly improve infection and associated management of insect pests with biological control in the subterranean environment. We are investigating this hypothesis.

## Methods

### Behavioral Plasticity after Volatile Exposure

*Steinernema diaprepesi* and *Heterorhabtidis indica* infective juvenile responses to pregeijerene, D-limonene, E -*β* caryophyllene, and controls were investigated in both exposed and nonexposed nematodes. Nonexposed infective juveniles were taken directly from culture flasks no more than three weeks after emerging from *Galleria mellonella* larvae and collection on White traps^22^,^23^. Exposed infective juveniles were prepared by placing approximately 300 infective juveniles in vials containing either pregeijerene, D-limonene, or E*-β* caryophyllene at 8 *μg*/*ml* for a duration of 24 hours.

Infective juvenile responses were monitored in two choice assays consisting of two 17ml glass jars connected by a three centimeter polypropylene tube^17^,^24^. Individual assays were filled with sand at 10% moisture by volume. Treatments (300 *ng* of either pregeijerene, D-limonene, E-*β* caryophyllene, or solvent control) were added to filter paper then placed in the olfactometer opposite a blank control. After 24 hours, responding nematodes were extracted from the sand in each opposing glass jar using Baermann funnels.

Four replications were conducted for each combination of exposed and nonexposed cohorts for each volatile (pregeijerene, D-limonene, and E-*β* caryophyllene) and each species (*S. diaprepesi* and *H. indica*). Mean numbers of responding infective juveniles for each combination were subjected to an exact binomial test. The relationship between change in response after exposure and initial nonexposed treatment preference across species was investigated with linear regression (bootstrapped 1000 times) for those assays where treatment matched exposure and for those assays where treatment did not match exposure. Data were normal (by investigation with Quantile-Quantile plots and interrogation with the Shapiro-Wilk test).

### Learning and Memory

To quantify the effect of the interaction between exposure time and postexposure time on behavioral plasticity, a D-optimal exposure x postexposure response surface design able to detect quadratic curvature for both factors was developed using Design Expert (version 9.0.4.1; StatEase Inc.). To this design, several additional points were added to aid in estimating model terms and lack of fit. Both factors (exposure, postexposure) were continuous and varied from 1 to 48 hours.

As dictated by the experimental design, *S. diaprepesi* infective juveniles were exposed to *α*-pinene from 1 to 48 hours by placing approximately 2500 infective juveniles in glass vials containing 5 *ml* water to which 5 *μl* of 1 *μg*/*μl α*-pinene in pentane was added. After the allotted exposure time, the infective juveniles were placed on a fine mesh sieve (400 mesh), washed gently with water, then placed in a clean glass vial (with no additional chemical treatment) until the postexposure time elapsed and they were ready for bioassays.

Similar to the behavioral plasticity trials above, two choice assays consisting of inverted T-Tubes constructed from 1.5 inch diameter PVC pipe were used to determine nematode attraction to *α*-pinene. Individual assays were filled with washed autoclaved sand at 12% moisture by volume after placing filter paper treated with either 10 *μl* of pentane (blank arm) or 10 *μl* of 10 *ng*/ *μl α*-pinene in pentane at opposing ends of the olfactometer. Infective juveniles were applied to the central orifice of the olfactometer at the end of their allotted post exposure time. After 24 hours, responding nematodes were extracted from the sand in each PVC cap using Baermann funnels.

A preference index (*I*_*p*_) for *α*-pinene was constructed using the responses from each olfactometer to facilitate comparisons across individual runs. This index better resolves differences in *α*-pinene preference and is constructed as the ratio between the numbers of nematodes responding to *α*-pinene and the number responding to the blank arm. An index of one denotes no preference, indices below one indicate repellence, and indices above one denote preference for *α*-pinene. All models from single factor to two factor quadratic polynomials were considered; models were selected based on low *P* values, high *R*^2^, and nonsignificant lack of fit. After passing initial inspection, models were inspected to ensure parsimony and adherence to assumptions of normality and homoscedasticity.

### Social Behavioral Plasticity

Similar to the learning and memory trials above, two choice assays consisting of inverted T-Tubes constructed from 1.5 inch diameter PVC pipe were used to determine nematode attraction to *α*-pinene. Individual assays were filled with washed autoclaved sand at 12% moisture by volume after placing filter paper treated with either 10 *μl* of pentane (blank control) or 10 *μl* of 10 *ng*/*μl α*-pinene in pentane at opposing ends of the olfactometer. Infective juveniles were applied to the central orifice of the olfactometer. After 24 hours, responding nematodes were extracted from the sand in each PVC cap using Baermann funnels.

Initially, preferences of exposed and nonexposed *S. diaprepesi* and *H. indica* to *α*-pinene were determined. Nonexposed nematodes were taken directly from culture flasks no more than two weeks after emerging from *G. mellonella* larvae and collection on White traps^22^. Nematodes were exposed for 48 hours by placing approximately 2500 infective juveniles in glass vials containing 5 ml water to which 5 *μl* of 1 *μg*/*μl α*-pinene in pentane was added.

Secondarily, 100 *S. diaprepesi* infective juveniles were exposed to 1 *μg*/*ml α*-pinene for 48 hours in 5 ml of water. After 48 hours, the exposed *S. diaprepesi* were added to olfactometers containing opposing treatments of *α*-pinene and blank control. Immediately following addition of exposed *S. diaprepesi*, 2500 nonexposed *H. indica* were added to the same olfactometers. Four replications were conducted for each independent combination of exposed and nonexposed cohorts of each species. Six replications were conducted for each simultaneous combination of exposed and nonexposed cohorts of each species. Numbers of responding individuals were converted to treatment response to facilitate comparisons across trials; data were normal by investigation with Quantile-Quantile plots and interrogation with the Shapiro-Wilk test.

## Additional Information

**How to cite this article**: Willett, D. S. *et al.* Social Networks of Educated Nematodes. *Sci. Rep.* **5**, 14388; doi: 10.1038/srep14388 (2015).

## Figures and Tables

**Table 1:** Experimental design and results for the exposure x postexposure memory and learning trial.

Run ID	Exposure Time (hours)	Postexposure Time (hours)	Blank Response	*α*-pinene Response	Preference Index
1	1.0	22.4	507	244	0.481
2	48.0	48.0	650	411	0.632
3	48.0	32.3	246	102	0.415
4	23.1	48.0	522	505	0.967
5	33.8	8.1	496	751	1.514
6	1.0	22.4	604	348	0.576
7	19.6	1.0	227	390	1.718
8	1.0	1.0	883	384	0.435
9	1.0	1.0	560	412	0.736
10	1.0	48.0	963	1369	1.422
11	33.7	37.2	386	538	1.394
12	48.0	1.0	496	1131	2.280
13	48.0	1.0	728	1796	2.467
14	1.0	48.0	2230	876	0.393
15	48.0	48.0	290	527	1.817
16	24.5	24.5	204	181	0.887
17	1.0	48.0	912	647	0.709
18	24.5	24.5	539	546	1.013
19	13.2	14.2	382	285	0.746
20	1.0	1.0	973	678	0.697
21	47.5	16.7	297	855	2.879
22	24.5	24.5	395	385	0.975
23	48.0	1.0	447	677	1.515
24	11.6	35.3	384	300	0.781
25	24.5	24.5	729	518	0.711
26	48.0	48.0	322	419	1.301
27	24.5	24.5	507	181	0.357

The trial is a D-optimal exposure x postexposure response surface design able to detect quadratic curvature for both factors and was developed using Design Expert (version 9.0.4.1; StatEase Inc.). To this design, several additional points were added to aid in estimating model terms and lack of fit. Both factors (exposure, postexposure) were continuous and varied from 1 to 48 hours. *S. diaprepesi* responses to *α*-pinene were assayed in two choice, sand-filled olfactometers. The preference index was constructed as the ratio between the numbers of nematodes responding to *α*-pinene and the number responding to the blank arm.

**Table 2:** ANOVA results and diagnostic measures for a response surface exploring the interaction between exposure time to *α*-pinene and postexposure time in clean culture on a preference index (*I*_*p*_ for *α*-pinene).

Source	F	df	*P*	RC
Model	8.55	3	**0.0005**	
Exposure	17.15	1	**0.0004**	0.035
Postexposure	3.39	1	*0.0786*	0.00401
Exposure x Postexposure	5.03	1	***0.0348***	−0.00056
Lack of Fit	2.08	10	0.1075	
*R*^2^	0.53			
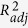	0.47			

*P* values in bold are significant at *α* = 0.05. *P* values in italics are significant at *α* = 0.1. RC values are regression coefficients.
